# Regional disparities in the availability of cancer clinical trials in Korea

**DOI:** 10.4178/epih.e2024006

**Published:** 2023-12-11

**Authors:** Jieun Jang, Wonyoung Choi, Sung Hoon Sim, Sokbom Kang

**Affiliations:** 1 Division of Clinical Research, Research Institute, National cancer center, Goyang, Korea; 2 Center for Clinical Trials, National Cancer Center, Goyang, Korea; 3 Division of Cancer Biology, Cancer Molecular Biology Branch, Research Institute, National Cancer Center, Goyang, Korea; 4 Department of Cancer Biomedical Science, National Cancer Center Graduate School of Cancer Science and Policy, Goyang, Korea; 5 Division of Clinical Research, Interventional Medicine Branch, Research Institute, National Cancer Center, Goyang, Korea; 6 Center for Breast Cancer, National Cancer Center, Goyang, Korea; 7 Center for Gynecologic Cancer, National Cancer Center, Goyang, Korea

**Keywords:** Clinical trial, Health services accessibility, Neoplasms, Healthcare disparities, Health inequities

## Abstract

**OBJECTIVES:**

Unequal access to cancer clinical trials is an important issue, given the potential benefits of participation for cancer patients. We evaluated regional disparities in access to cancer clinical trials in Korea.

**METHODS:**

From the Ministry of Food and Drug Safety database, we extracted 2,465 records of all cancer clinical trials approved between January 2012 and April 2023. To measure disparities in cancer clinical trial access, we calculated the ratio of clinical trials open to non-capital areas relative to those open to capital areas. We then analyzed temporal trends in this ratio, which we termed the trial geographical equity index (TGEI).

**RESULTS:**

Disparities in access to cancer clinical trials, as indicated by the TGEI, did not significantly improve during the study period (regression coefficient, 0.002; p=0.59). However, for phase II/III trials sponsored by global pharmaceutical companies, the TGEI improved significantly (regression coefficient, 0.021; p<0.01). In contrast, the TGEI deteriorated for trials initiated by investigators or those testing domestically developed therapeutics (regression coefficient, -0.015; p=0.05). Furthermore, the increasing trend of TGEI for phase II/III trials sponsored by global companies began to reverse after 2019, coinciding with the outbreak of coronavirus disease 2019 (COVID-19).

**CONCLUSIONS:**

Over the past decade, access to cancer clinical trials has improved in Korea, particularly for phase II/III trials evaluating therapeutics from global companies. However, this increase in accessibility has not extended to trials initiated by investigators or those assessing domestically developed therapeutics. Additionally, the impact of COVID-19 on disparities in clinical trial access should be closely monitored.

## INTRODUCTION

Equitable access to clinical trials for cancer treatments is crucial for multiple reasons. From the standpoint of developing new therapeutics, equitable access is essential to refine drug development and to ensure the generalizability of study results [1]. Moreover, clinical trial participation is often the sole avenue through which cancer patients can receive the most advanced care and benefit from the latest medical breakthroughs. This access offers numerous advantages, including improved outcomes, psychological benefits, and enhanced quality of life for those with cancer [1-6]. Thus, affording cancer patients equal opportunities to join clinical trials is an important concern, as it allows for equitable medical benefits and helps to eliminate disparities in cancer care [7]. Clinical trial participation is influenced by a range of factors. These include intrinsic patient characteristics, such as income, education, race, personal risk and gain, and fear, as well as extrinsic factors, such as cost and physician influence [1,8,9]. However, the greatest obstacle is the lack of available trials, a structural and systemic issue that prevents many cancer patients from taking part in clinical studies [9]. Consequently, assessing the equity of clinical trial access across regions is valuable in determining the fairness of medical care available to cancer patients.

In Korea, the Ministry of Food and Drug Safety (MFDS) oversees the regulation of clinical drug trial protocols and the approval of bioequivalence studies [10]. The past 20 years have been marked by an unprecedented increase in the number of clinical trials conducted in the country. In fact, the MFDS approved approximately 20 times the number of clinical trials in 2018 compared to 2000 [11]. Despite this remarkable growth in clinical trial activity, most trial sites are concentrated in the densely populated capital region [12]. Consequently, it is necessary to assess whether the availability of cancer clinical trials is adequate in areas outside the capital. Additionally, identifying which types of cancer clinical trials have contributed to either improving or worsening regional equity of access is instrumental in securing and enhancing the equitable distribution of cancer clinical trial opportunities in the future.

During the coronavirus disease 2019 (COVID-19) pandemic, the field of clinical research faced numerous challenges, such as halted recruitment and extended project timelines [13]. While conducting clinical trials in Korea has been difficult since the onset of the COVID-19 pandemic, the extent to which regional disparities in access to clinical trials have worsened remains underexplored.

Consequently, our objective was to assess the temporal evolution of regional disparities in access to cancer clinical trials within Korea. Specifically, we examined the variation in regional disparities in the availability of cancer clinical trials before and after the onset of COVID-19. Furthermore, we identified clinical trial types that exhibited an improvement in regional equity and contrasted them with those requiring more substantial efforts to decrease regional disparities, considering trial characteristics.

## MATERIALS AND METHODS

From the Korean MFDS database, we retrieved 9,424 records representing all clinical trials approved between January 2012 and April 2023. These records contained details such as the brands and generic names of the drugs, the phases of the trials, the origin of the pharmaceutical companies (either global or domestic), the dates of trial approval, the clinical trial sites, and the titles of the trials. The disease name was inferred from the title of each trial. Of the total, 2,465 clinical trials were identified that specifically focused on testing cancer treatments. These trials involved 109 trial sites.

Korea contains 17 first-tier administrative districts, corresponding to the Organization for Economic Cooperation and Development Territorial level 3 regions. These include Seoul (the capital city), 6 metropolitan cities, 1 special self-governing city, and 9 provinces. We divided these 17 administrative divisions into 2 groups: the capital area, which comprised the capital city, Incheon Province, and Gyeonggi Province, and the non-capital area, which consisted of the remaining divisions not part of the capital area. This distinction was made due to reported disparities in health outcomes between the seoul capital area and the other regions [14].

We classified all trials based on whether they were open to participants in the capital area and/or open to those in the non-capital area. To assess regional disparities in access to cancer clinical trials, we established a metric termed the trial geographical equity index (TGEI). This index represents the ratio of clinical trials available in a given region (here, the capital area) to those available in another region (in this case, the non-capital area). A TGEI value of 1 suggests that no disparity is present. In contrast, a TGEI value substantially different from 1 may indicate a disparity in access to clinical trials between the regions. We computed the TGEI annually, considering the approval year of each trial in the calculation. To determine temporal trends for disparities in access to cancer trials, we employed a linear regression model to estimate changes in the TGEI over time.

A subgroup analysis was conducted to evaluate the pattern of differences in access to cancer trials, considering variables such as the origin of the drug developer (domestic vs. global), the stage of the clinical trial (phase II/III vs. other phases), and the specific cancer sites targeted. Additionally, given that the COVID-19 pandemic may have influenced clinical trials through various mechanisms, including the reallocation of resources and interruptions in the supply chain, we posited that the pandemic might have altered the temporal pattern of these disparities. To investigate this hypothesis, we compared the trends from the pre-pandemic period (2012-2019) to those of the post-pandemic period (2020-2023).

All statistical analyses were performed using R version 4.3.0 (R Foundation for Statistical Computing, Vienna, Austria), and a 2-sided alpha error of less than 0.05 was considered to indicate statistical significance.

### Ethics statement

This study was exempt from review by the institutional review board since it is not the research involving human subjects.

## RESULTS

From January 2012 to April 2023, a total of 2,465 cancer clinical trials received approval in Korea. More than 97% of these trials were available in the capital area, with 1,038 trials open to both the capital and non-capital areas, and 1,362 trials exclusively open to the capital area. Only 65 trials, or 2.6%, were available solely in non-capital areas (Table 1). This disparity suggests that cancer patients residing in the capital area had the opportunity to participate in 97.4% of the cancer clinical trials (2,400 of 2,465 trials) conducted during the study period. Conversely, those living in non-capital areas had access to only 44.7% of the trials (1,103 of 2,465). When examining phase II/III trials specifically, cancer patients in the capital area could participate in 98.9% of these trials (1,147 of 1,160 trials), while their counterparts in non-capital areas had access to 58.7% (681 of 1,160 trials). For trials testing pharmaceuticals from global companies, non-capital trial sites were included in 48% of cases, yet 99% of these trials were open to sites in the capital. Similarly, for trials testing drugs developed by domestic companies, non-capital sites were included in 40%, while 93% were open to sites in the capital.

**Figure 1.**
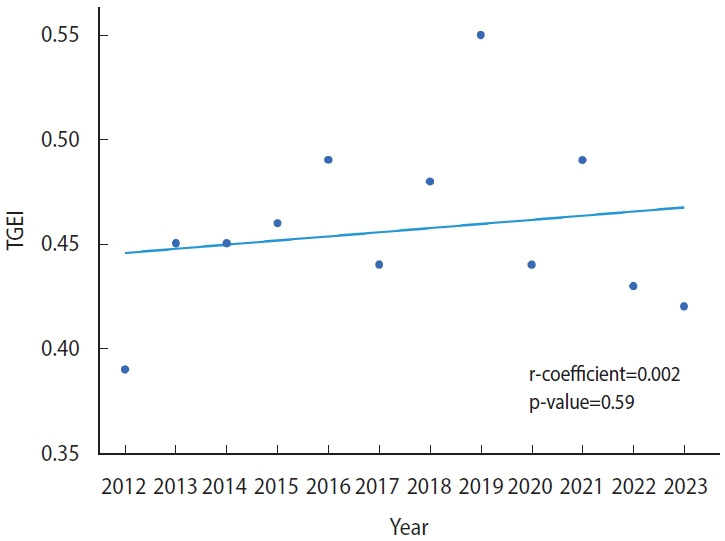
Temporal trend in the trial geographical equity index (TGEI) from 2012 to 2023 in Korea.

Table 2 summarizes the trend in disparity regarding access to clinical trials over the study period. The TGEI did not exceed 0.5 at any point between 2012 and 2023, except for a single occurrence in 2019. This suggests that the substantial difference in access to cancer trials has not improved over the past decade. Furthermore, analysis using a linear regression model revealed no evidence of a reduction in this disparity (regression coefficient, 0.002; p= 0.59; Figure 1).

**Figure 2.**
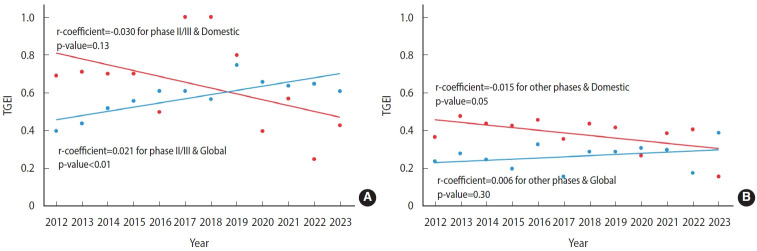
Temporal trend in the trial geographical equity index (TGEI) from 2012 to 2023 in Korea, considering the type of developer for the therapeutic. (A) Phase II/III trials. (B) Phases other than II/III.

However, the subgroup analysis revealed a sector in which regional disparities meaningfully decreased. Specifically, a significant improvement in the TGEI was observed for phase II/III trials testing therapeutics from global pharmaceutical companies (regression coefficient, 0.021; p< 0.01; Figure 2A and Table 3). In contrast, the TGEI significantly declined for trials not in phase II/III and those testing products from domestic pharmaceutical companies, including investigator-initiated trials (regression coeffiient, -0.015; p= 0.05; Figure 2B and Table 3). No significant temporal change in the TGEI was observed for phase II/III trials of domestically developed therapeutics (p= 0.13; Figure 2A and Table 3), or for clinical trials other than phase II/III trials that tested therapeutics from global companies (p= 0.30; Figure 2B and Table 3).

**Figure 3.**
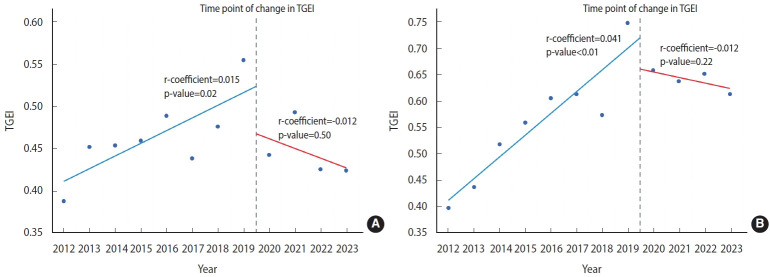
Disparity in temporal trends of the trial geographical equity index (TGEI) before and after onset of the coronavirus disease 2019 pandemic in Korea. (A) Overall cancer clinical trials. (B) Phase II/III trials evaluating cancer therapeutics developed by global companies.

Considering our hypothesis that the COVID-19 pandemic may have influenced clinical research, we also investigated temporal variations in the TGEI before and after the onset of the pandemic. As depicted in Figure 3A, our analysis revealed an upward trend in TGEI from 2012 to 2019, indicating a potential improvement in regional equity of access to cancer clinical trials during that timeframe (regression coefficient, 0.015; p= 0.02). However, the TGEI decreased from 2020 to 2023 (regression coefficient, -0.012; p= 0.50), suggesting a possible deterioration in regional equity of cancer trial access (Figure 3A). A comparable trend was noted for phase II/III cancer clinical trials for drugs developed by global companies, with a significant increase in the TGEI between 2012 and 2019 (regression coefficient, 0.041; p< 0.01) and a decrease in TGEI after 2019 (regression coefficient, -0.012; p= 0.22; Figure 3B).

Based on our analysis of targeted cancer sites, we found that cancer clinical trials for lymphoma, lung cancer, bladder cancer, and prostate cancer exhibited TGEIs greater than 0.5 between 2012 and 2023 (Supplementary Material 1). In contrast, the TGEIs for other cancer sites, including the colon and rectum, corpus uteri, esophagus, thyroid, gallbladder, and ovaries, and other solid type cancers were below 0.4. These findings indicate that the extent of regional disparities in trial access may vary by cancer site. Specifically, when examining phase II/III trials for therapies developed by global pharmaceutical companies, we noted significant improvements in regional disparities for breast cancer, lung cancer, lymphoma, and kidney cancer. The corresponding regression coefficients and p-values were as follows: 0.027 (p= 0.05) for breast cancer; 0.032 (p= 0.03) for lung cancer; 0.053 (p= 0.02) for lymphoma; and 0.079 (p=0.02) for kidney cancer (Supplementary Materials 2 and 3).

## DISCUSSION

In this study, our objective was to examine regional differences in the availability of cancer clinical trials in Korea over the past decade. Our findings indicate that the proportion of cancer clinical trials available in non-capital areas has never exceeded 60% of those available in the capital region, and no significant progress was evident in regional equity of access to clinical trials between 2012 and 2023. However, for phase II/III trials involving therapeutics developed by global companies, we observed a significant reduction in regional disparities in trial accessibility. Despite this, it appears that this progress regarding phase II/III trials for therapeutics from global companies may have stalled after 2019.

To date, few studies have explored regional differences in the availability of cancer clinical trials at a national level [15,16]. A recent investigation assessed these regional disparities within Korea by utilizing the MFDS database, an approach like that of our study [15]. That research revealed a concentration of cancer clinical trials in Seoul, the capital city, with a decreasing number of trials available in the surrounding capital region, metropolitan cities, and then provincial areas, echoing our findings. However, that study did not address the temporal evolution of these regional disparities or the progress made towards equalizing access to trials across regions. Therefore, we believe that our study’s insights into the trends in regional disparities contribute new and meaningful information regarding the accessibility of cancer clinical trials in Korea.

A separate study performed in India similarly investigated regional disparities in access to cancer clinical trials [16]. In that research, the investigators created an index to measure the accessibility of clinical trials in each region by determining the ratio of participants enrolled over a 1-year recruitment period to the incidence of cancer in each state. The findings revealed state-by-state variations in the opportunities available for cancer patients to participate in clinical trials within their regions of residence. Notably, the extent of regional disparities was comparatively low for trials sponsored by the industry. This observation aligns with the results of our study, which indicated that regional disparities in clinical trial access were significantly reduced only in phase II/III trials that had a high percentage of sponsor-initiated trials.

Establishing a clinical trial site requires infrastructure equipped with essential resources, such as human capital, financial backing, and facilities crucial for trial oversight and authorization [17]. In Korea, over half of the approved clinical trial sites are in the capital region, where more than half of the population also resides [18,19]. Consequently, conducting clinical trials in the capital area is comparatively efficient, thanks to the ample infrastructure and human resources as well as the adequate pool of potential participants. Moreover, since the national healthcare system imposes no restrictions on the selection of and access to medical institutions [20], a considerable number of cancer patients in Korea travel to the capital region for treatment [21,22]. This overall landscape fosters geographical disparities in the availability of cancer trials.

Although no overall progress was observed in the regional equity of access to cancer clinical trials in Korea, the disparity significantly improved for phase II/III cancer therapeutic trials conducted by global pharmaceutical companies. This improvement may be attributed to the nature of these trials. Phase II/III trials, which evaluate therapeutics developed by global companies, typically possess adequate resources and budgets to establish numerous trial sites, including those in smaller hospitals beyond the capital region. This expansion could contribute to a reduction in regional disparities in the availability of trials. Consequently, strategic initiatives from large global pharmaceutical companies will likely play an increasingly pivotal role in mitigating geographical disparities in access to cancer trials. In contrast, trials conducted by domestic companies or individual investigators often face constraints due to limited budgets and resources [23]. This limitation may account for the lack of impact of these clinical trials on reducing geographic disparities in cancer clinical trials. Despite these challenges, it is encouraging to observe that major pharmaceutical companies are endeavoring to address regional disparities in access to cancer trials. Nevertheless, concerns persist that the mitigation of these disparities is contingent upon the strategies and circumstances of these large global pharmaceutical companies.

In this study, we also observed that the improvement in regional disparities in clinical trial access ceased after 2019. This interruption of the positive trend in geographic disparities in access may be linked to the emergence of COVID-19. With the onset of the pandemic, the execution of clinical trials faced numerous obstacles, including difficulties in maintaining participants’ scheduled study visits, organizing site initiation meetings, and securing database access [24]. This may have led to a decrease in available resources or a consolidation of trial sites, exacerbating regional disparities in access to cancer clinical trials. Nonetheless, the COVID-19 pandemic has highlighted the importance of conducting virtual trials through technologies such as telemedicine or remote monitoring [25]. It will be intriguing to determine whether this shift towards virtual trials will help to reduce regional disparities in access to clinical trials in the near future.

Improvements in regional disparities regarding the availability of cancer clinical trials have not been uniformly observed across all tumor types. Notably, only lung cancer, breast cancer, lymphoma, and kidney cancer have seen significant reductions in these disparities. These cancers are characterized by either large market sizes or high unmet medical needs, factors that contribute substantially to pharmaceutical sales [26]. Consequently, this trend could introduce a new form of inequity across tumor types, posing a potential problem. Therefore, it is imperative that additional efforts be made to address geographic disparities in clinical trials for cancers that have a smaller market presence or are considered rare.

While we have highlighted regional disparities in cancer clinical trial access by demonstrating the greater number of trials in the capital region compared to non-capital areas, our method has limitations. Namely, it merely contrasts the quantity of trials without considering the incidence or prevalence of cancer in each region. Although the capital area hosts most cancer clinical trials, it also represents over 50% of Korea’s total population and cancer cases [19,27]. Consequently, a simple comparison of the number of trials between the capital and non-capital areas may not provide an accurate assessment of regional equity in clinical trial access. The MFDS database we utilized did not include data on the planned or actual enrollment numbers for each trial, preventing us from incorporating participant numbers into our analysis of regional disparities in trial access. Adjusting the count of accessible clinical trials for the number of cancer patients in each region allows for a direct comparison of the possibility of patient participation in trials between regions. However, if we use the level of access to and participation in clinical trials within each region as a measure of regional equity, the absolute number of trials available in each region may be a more suitable indicator for assessing equity. Moreover, if an index that accounts for patient numbers is employed to evaluate regional equity, regions with fewer cancer patients might appear to have adequate trial access, despite having many fewer actual opportunities for participation.

This study also has limitations concerning trial classification. We divided the clinical trials into 2 categories based on their phase: phase II/III and all other phases. The latter category encompasses investigator-initiated trials (IITs), phase I, combined phase I and II, phase I and III, and extended trials, along with trials of uncertain phase. In this study, IITs represent over 53.3% of the trials not in phase II/III, and it is possible that this group may include preclinical or post-marketing surveillance trials. Unfortunately, the MFDS database does not provide detailed information about the types of IITs, preventing us from excluding preclinical or post-marketing surveillance trials from our analysis.

The findings of this study highlight a scarcity of clinical trials in non-capital areas of Korea, a factor previously identified as a substantial obstacle to achieving equitable access to clinical trials [9]. Increasing awareness of the regional imbalances in clinical trial access is vital to improving the equity of availability. Furthermore, it is essential to identify the barriers impeding the initiation of clinical trials in non-capital areas and to collaborate with healthcare providers, governmental bodies, and trial sponsors to devise strategies that are financially well-supported [28].

## Figures and Tables

**Table 1.** Characteristics of cancer clinical trials approved from 2012-2023 in Korea

Characteristics	Total	Trials open to both capital and non-capital areas	Trials open to capital area only	Trials open to non-capital area only	p-value
No. of trials	2,465	1,038	1,362	65	
Phase					<0.01
II/III	1,160 (100)	668 (57.6)	479 (41.3)	13 (1.1)	
Others	1,305 (100)	370 (28.3)	883 (67.7)	52 (4.0)	
Developer of therapeutic					<0.01
Global company	1,582 (100)	746 (47.2)	828 (52.3)	8 (0.5)	
Domestic company	883 (100)	292 (33.1)	534 (60.4)	57 (6.5)	

Values are presented as number (%).

**Table 2.** Ratio of the number of cancer clinical trials open to non-capital and capital areas from 2012-2023 in Korea

Year	Trials open to non-capital area	Trials open to capital area	TGEI^1^
2012	69	178	0.39
2013	66	146	0.45
2014	82	181	0.45
2015	107	233	0.46
2016	84	172	0.49
2017	102	233	0.44
2018	107	225	0.48
2019	107	193	0.55
2020	119	269	0.44
2021	130	264	0.49
2022	94	221	0.43
2023	36	85	0.42

TGEI, trial geographical equity index.

1 Ratio of the number of trials open to the non-capital area relative to the number open to the capital area.

**Table 3.** Ratio of the number of cancer clinical trials open to non-capital and capital areas from 2012-2023 in Korea, considering type of therapeutic developer and trial phase

Year	Phase II/III trials						Trials other than phase II/III					
	Trials testing therapeutic from a global company			Trials testing therapeutic from a domestic company			Trials testing therapeutic from a global company			Trials testing therapeutic from a domestic company		
	Non-capital^1^	Capital^2^	TGEI^3^	Non-capital^1^	Capital^2^	TGEI^3^	Non-capital^1^	Capital^2^	TGEI^3^	Non-capital^1^	Capital^2^	TGEI^3^
2012	25	63	0.40	11	16	0.69	7	29	0.24	26	70	0.37
2013	31	71	0.44	12	17	0.71	7	25	0.28	16	33	0.48
2014	44	85	0.52	7	10	0.70	9	36	0.25	22	50	0.44
2015	62	111	0.56	7	10	0.70	9	45	0.20	29	67	0.43
2016	43	71	0.61	3	6	0.50	14	43	0.33	24	52	0.46
2017	57	93	0.61	7	7	1.00	8	49	0.16	30	84	0.36
2018	55	96	0.57	4	4	1.00	13	45	0.29	35	80	0.44
2019	65	87	0.75	4	5	0.80	10	35	0.29	28	66	0.42
2020	71	108	0.66	4	10	0.40	23	74	0.31	21	77	0.27
2021	81	127	0.64	4	7	0.57	20	66	0.30	25	64	0.39
2022	58	89	0.65	4	16	0.25	12	67	0.18	20	49	0.41
2023	19	31	0.61	3	7	0.43	11	28	0.39	3	19	0.16

TGEI, trial geographical equity index.

1 Cancer clinical trials open to non-capital area.

2 Cancer clinical trials open to capital area.

3 Ratio of the number of trials open to the non-capital area relative to the number open to the capital area.

## Supplementary materials

Supplementary Material 1. Ratio of the number of cancer clinical trials open to non-capital area relative to the number open to the capital area from 2012-2023 in Korea, considering cancer site

Supplementary Material 2. Annual change of the trial geographical equity index (TGEI) in access to phase II/III cancer clinical trials to test therapeutic developed by global company from 2012-2023 in Korea, considering cancer site.

Supplementary Material 3. Temporal trend in the trial geographical equity index (TGEI) from 2012 to 2023 in Korea, considering trial phase, type of therapeutic developer and cancer site
