# Supplementary material for: Regional disparities in the availability of cancer clinical trials in Korea
Source: Epidemiol Health. 2023 Dec 11;46:e2024006. doi: 10.4178/epih.e2024006 (PMC11040215; doi:10.4178/epih.e2024006)
Supplement: Supplementary Material 1. — Ratio of the number of cancer clinical trials open to non-capital area relative to the number open to the capital area from 2012-2023 in Korea, considering cancer site [file epih-46-e2024006-Supplementary-1.docx]

**Supplementary Material 1. Ratio of the number of cancer clinical trials open to non-capital area relative to the number open to the capital area from 2012-2023 in Korea, considering cancer site**

| **Cancer Site/type** | **Trials open to non-capital area** | **Trials open to capital area** | **TGEI^1^** |
| --- | --- | --- | --- |
| Lymphoma | 111 | 184 | 0.60 |
| Lung | 235 | 410 | 0.57 |
| Bladder | 15 | 27 | 0.56 |
| Prostate | 40 | 76 | 0.53 |
| Cervix uteri | 10 | 20 | 0.50 |
| Others | 96 | 191 | 0.50 |
| Liver | 57 | 120 | 0.48 |
| Kidney | 14 | 29 | 0.48 |
| Pancreas | 29 | 64 | 0.45 |
| Leukemia | 66 | 147 | 0.45 |
| Breast | 112 | 254 | 0.44 |
| Stomach | 69 | 159 | 0.43 |
| Colorectal | 33 | 88 | 0.38 |
| Corpus uteri | 5 | 13 | 0.38 |
| Esophagus | 9 | 24 | 0.38 |
| Solid type | 167 | 481 | 0.35 |
| Thyroid | 3 | 9 | 0.33 |
| Gallbladder^2^ | 16 | 50 | 0.32 |
| Ovary | 16 | 54 | 0.30 |

TGEI, trial geographical equity index

1. Ratio of the number of trials open to non-capital area relative to the number of open to the capital area
2. Includes the gallbladder and other/unspecified parts of the biliary tract
