# Supplementary material for: Regional disparities in the availability of cancer clinical trials in Korea
Source: Epidemiol Health. 2023 Dec 11;46:e2024006. doi: 10.4178/epih.e2024006 (PMC11040215; doi:10.4178/epih.e2024006)
Supplement: Supplementary Material 2. — Annual change of the trial geographical equity index (TGEI) in access to phase II/III cancer clinical trials to test therapeutic developed by global company from 2012-2023 in Korea, considering cancer site. [file epih-46-e2024006-Supplementary-2.docx]

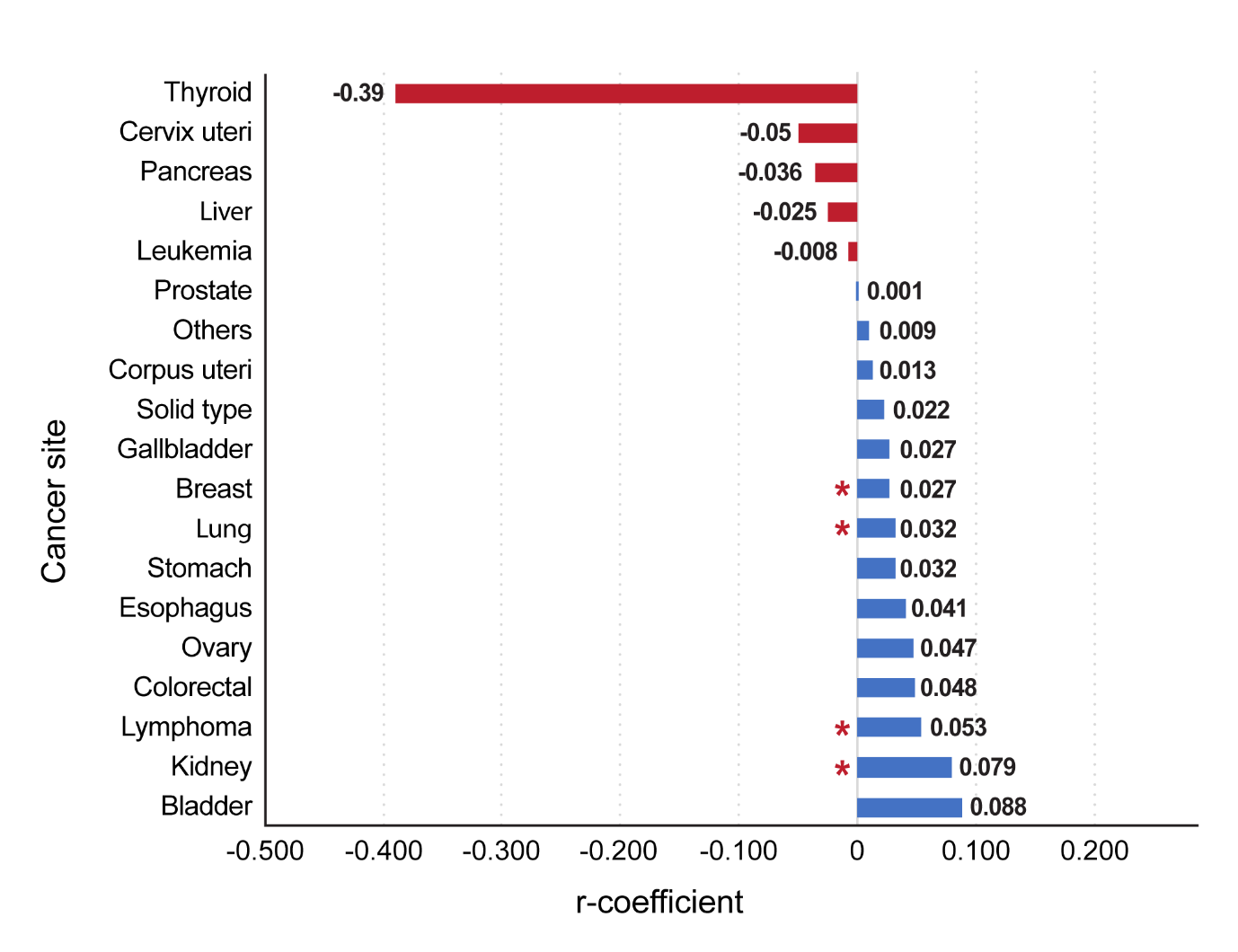


**Supplementary Material 2. Annual change of the trial geographical equity index (TGEI) in access to phase II/III cancer clinical trials to test therapeutic developed by global company from 2012-2023 in Korea, considering cancer site.** Asterisk (*) mark indicates a statistically significant (*p*-value <0.05) linear regression coefficient.
