# Supplementary material for: Regional disparities in the availability of cancer clinical trials in Korea
Source: Epidemiol Health. 2023 Dec 11;46:e2024006. doi: 10.4178/epih.e2024006 (PMC11040215; doi:10.4178/epih.e2024006)
Supplement: Supplementary Material 3. — Temporal trend in the trial geographical equity index (TGEI) from 2012 to 2023 in Korea, considering trial phase, type of therapeutic developer and cancer site [file epih-46-e2024006-Supplementary-3.docx]

**Supplementary Material 3. Temporal trend in the trial geographical equity index (TGEI) from 2012 to 2023 in Korea, considering trial phase, type of therapeutic developer and cancer site**

| **Site/type** | **Overall** | | **Phase II/III testing global company therapeutic** | | **Other phases testing domestic company**  **therapeutic** | |
| --- | --- | --- | --- | --- | --- | --- |
|  | **Regression coefficient** | ***p*-value** | **Regression coefficient** | ***p*-value** | **Regression coefficient** | ***p*-value** |
| Lung | 0.017 | 0.05 | 0.032 | 0.03 | -0.008 | 0.70 |
| Liver | -0.023 | 0.15 | -0.025 | 0.27 | -0.010 | 0.76 |
| Colorectal | 0.025 | 0.30 | 0.048 | 0.22 | 0.013 | 0.63 |
| Stomach | -0.006 | 0.54 | 0.032 | 0.13 | 0.010 | 0.55 |
| Pancreas | -0.044 | 0.02 | -0.036 | 0.30 | -0.020 | 0.61 |
| Gallbladder^1^ | -0.011 | 0.78 | 0.027 | 0.65 | -0.069 | 0.27 |
| Breast | 0.005 | 0.08 | 0.027 | 0.05 | -0.037 | 0.14 |
| Prostate | -0.014 | 0.59 | 0.001 | 0.08 | -0.012 | 0.80 |
| Lymphoma | 0.006 | 0.65 | 0.053 | 0.02 | -0.002 | 0.95 |
| Leukemia | 0.018 | 0.17 | -0.008 | 0.70 | 0.073 | 0.05 |
| Thyroid | -0.118 | 0.02 | -0.390 | 0.07 | -0.108 | 0.01 |
| Kidney | 0.027 | 0.34 | 0.079 | 0.02 | 0.024 | 0.88 |
| Bladder | 0.011 | 0.82 | 0.088 | 0.13 | -0.109 | 0.09 |
| Cervix uteri | -0.010 | 0.84 | -0.050 | 0.27 | -0.119 | 0.24 |
| Corpus uteri | -0.029 | 0.43 | 0.013 | 0.60 | N/A | N/A |
| Ovary | 0.037 | 0.03 | 0.047 | 0.11 | 0.046 | 0.32 |
| Esophagus | -0.025 | 0.43 | 0.041 | 0.57 | -0.097 | 0.10 |
| Solid type | -0.005 | 0.34 | 0.022 | 0.12 | -0.050 | 0.01 |
| Others | 0.002 | 0.90 | 0.009 | 0.73 | -0.007 | 0.81 |

N/A, not available

1. Includes the gallbladder and other/unspecified parts of the biliary tract.
